# Biomarker discovery: quantification of microRNAs and other small non-coding RNAs using next generation sequencing

**DOI:** 10.1186/s12920-015-0109-x

**Published:** 2015-07-01

**Authors:** Juan Pablo Lopez, Alpha Diallo, Cristiana Cruceanu, Laura M. Fiori, Sylvie Laboissiere, Isabelle Guillet, Joelle Fontaine, Jiannis Ragoussis, Vladimir Benes, Gustavo Turecki, Carl Ernst

**Affiliations:** McGill Group for Suicide Studies (MGSS), Douglas Mental Health University Institute, McGill University, Frank B Common Pavilion, Room F-2101.2, 6875 LaSalle Boulevard, Montreal, QC H4H 1R3 Canada; Department of Human Genetics, McGill University, Montreal, QC Canada; McGill University and Genome Quebec Innovation Centre, Montreal, QC Canada; European Molecular Biology Laboratory (EMBL), Genomics Core Facility, Heidelberg, Germany

**Keywords:** Biomarker, microRNA, Small non-coding RNA, Next-generation sequencing, Small RNA sequencing, Whole-blood, Brain, Heart, Liver, Clinical samples

## Abstract

**Background:**

Small ncRNAs (sncRNAs) offer great hope as biomarkers of disease and response to treatment. This has been highlighted in the context of several medical conditions such as cancer, liver disease, cardiovascular disease, and central nervous system disorders, among many others. Here we assessed several steps involved in the development of an ncRNA biomarker discovery pipeline, ranging from sample preparation to bioinformatic processing of small RNA sequencing data.

**Methods:**

A total of 45 biological samples were included in the present study. All libraries were prepared using the Illumina TruSeq Small RNA protocol and sequenced using the HiSeq2500 or MiSeq Illumina sequencers. Small RNA sequencing data was validated using qRT-PCR. At each stage, we evaluated the pros and cons of different techniques that may be suitable for different experimental designs. Evaluation methods included quality of data output in relation to hands-on laboratory time, cost, and efficiency of processing.

**Results:**

Our results show that good quality sequencing libraries can be prepared from small amounts of total RNA and that varying degradation levels in the samples do not have a significant effect on the overall quantification of sncRNAs via NGS. In addition, we describe the strengths and limitations of three commercially available library preparation methods: (1) Novex TBE PAGE gel; (2) Pippin Prep automated gel system; and (3) AMPure XP beads. We describe our bioinformatics pipeline, provide recommendations for sequencing coverage, and describe in detail the expression and distribution of all sncRNAs in four human tissues: whole-blood, brain, heart and liver.

**Conclusions:**

Ultimately this study provides tools and outcome metrics that will aid researchers and clinicians in choosing an appropriate and effective high-throughput sequencing quantification method for various study designs, and overall generating valuable information that can contribute to our understanding of small ncRNAs as potential biomarkers and mediators of biological functions and disease.

**Electronic supplementary material:**

The online version of this article (doi:10.1186/s12920-015-0109-x) contains supplementary material, which is available to authorized users.

## Background

There is significant interest in the prediction and early detection of disease through the analysis of biological markers, or biomarkers, which have the potential to significantly improve clinical outcomes [1, 2]. Biomarkers are defined as any molecule derived from a biological sample that can indicate current disease status, evaluate progression of the disease, or assess potential responsiveness to a particular medication [3]. Biomarkers come in many forms including DNA mutations, proteins, and messenger RNA (mRNA) transcripts [4]. For example, ratios of aspartate/alanine aminotransferase are used as a reliable biomarker for liver fibrosis [5], protein levels of S100-beta are used as a biomarker of treatment response for malignant melanoma [6], while mutations of the genes BRCA1 and BRCA2 are well known biomarkers predicting the development of breast cancer [7]. DNA methylation is also a well-studied biomarker [8–10]. Though not a focus of the current report, methylated cytosine residues have been associated with several diseases, including cancer and neurological disorders [11].

Over the years, non-coding RNAs (ncRNAs) have become the focus of biomarker research, an approach that has been favorably used in the investigation of response to treatment for several medical conditions. There are several types of ncRNAs, of which microRNAs (miRNAs) are the best known and the most frequently assessed for their potential role as biomarkers. MiRNAs have been proposed as molecular biomarkers in cancer [12], liver and cardiovascular disease [13, 14], and central nervous system disorders [15–18], among many others [19–22]. MiRNAs are small ncRNAs molecules that follow a well characterized biogenesis pathway that includes processing through the DGCR8/ DROSHA, Exportin-5, Dicer and RISC molecular complexes [23]. Through post-transcriptional activity, these small, single-stranded, 19–25-base RNA transcripts regulate the expression of numerous genes. Binding of the miRNA to the complementary sequence of a target mRNA relies on recognition of the seed region, the 2–8 nucleotides located at the 3′end of the miRNA, which leads to either mRNA degradation or translational repression [19, 21, 24].

Other ncRNA species such as PIWI-interacting RNAs (piRNAs), small nucleolar RNAs (snoRNAs), small nuclear RNAs (snRNAs) and long non-coding RNAs are also gaining support as key components of cellular regulation [19, 25], and thus might be potentially assessed as biomarkers of disease. PiRNAs are small ncRNAs of 24–31 nt length. In contrast to miRNAs, these are Dicer-independent and interact with the PIWI subfamily of Argonaute proteins involved in the regulation of genome stability [26, 27]. PIWI proteins are involved in gene regulation through RNA degradation and have been linked to DNA methylation [28]. In addition, piRNAs have been reported as potential biomarkers for bladder [29], breast [30], and gastric [31] cancers. SnoRNAs are key components of the small ribonucleoproteins (snoRNPs) which are responsible for sequence-specific 2′-*O*-Methylation of ribosomal RNA (rRNA) [32]. SnoRNAs have been shown to participate in post-transcriptional regulation of rRNA by targeting snoRNPs in the nucleus [33]. In addition, snoRNAs have been proposed as potential biomarkers for several forms of human cancers [34–36]. Long non-coding RNAs are another class of ncRNAs that have gained a lot of attention recently as potential biomarkers [37–40]. They comprise a heterogeneous group of ncRNAs larger than 200 nt, which includes long non-coding RNAs (lncRNAs), large intergenic non-coding RNAs (lincRNAs) and transcribed ultraconserved regions (T-UCRs), among others [25]. LncRNAs are known to regulate DNA methylation by recruiting chromatin remodeling complexes [41]. LincRNAs have been associated with active transcription in regions of transcriptional elongation [42]. Finally, while the function of T-UCRs is still unknown, they have been demonstrated to interact with microRNAs and might have a role in the development of disease [43]. T-UCRs have been recently postulated as potential diagnostic and prognostic biomarkers in colorectal cancer patients [44].

While any ncRNA is a putative biomarker, miRNAs have received the most attention because they possess several features that render them especially powerful : (1) they are highly conserved, and evolutionary complexity correlates with miRNA complexity, which suggests an important biological function; (2) there are a relatively small number of individual miRNAs with a large dynamic range of expression; (3) they are secreted into circulation and can be measured in all biological fluids; (4) they are not easily degraded and are thus highly stable in clinical samples; (5) they are involved in pathway regulation, as one miRNA can target many genes, and a single gene can be regulated by many different miRNAs; (6) miRNAs show tissue and cell specific expression profiles; and (7) there is a large body of literature supporting their role in the pathophysiology of disease [45].

Most ncRNA quantification studies performed to date rely on qRT-PCR, *in situ* hybridization, or microarray techniques. These methods have several strengths, but also contain some important limitations. These include: the number of miRNA molecules that can be analyzed simultaneously, the amount of RNA required for the analysis of multiple targets, the quality and source of the RNA, the sensitivity of detection, and the need for previous knowledge of targets [46]. Next generation sequencing (NGS) provides researchers with a powerful tool for the detection of RNA molecules in biological samples. NGS offers methodological advantages such as increased throughput, decreased RNA input, consistency and quality of data, higher detection depth, analysis of all RNA populations, and discovery of novel molecules. Furthermore, length of protocols, sequencing time, and prices are continuously dropping, making NGS an ideal tool for biomarker research [47].

In terms of clinical utility, blood is a reliable and non-invasive source of biological tissue that reflects different stages of disease. Blood samples are relatively easy to collect and can be stored over long periods of time without having a significant effect on the levels of miRNAs and other ncRNAs in whole-blood, plasma or serum [48]. As biomarker research using ncRNAs is still in its infancy, there is no consensus yet on the best source of blood cells for the study of disease. Some studies suggest that whole-blood, peripheral blood mononuclear cells (PBMCs), or white blood cells (WBCs) are good sources to explore ncRNAs which have been secreted into circulation. In addition, these cells can provide important information on inflammatory states [49]. On the other hand, some argue that plasma or serum are optimal to investigate ncRNAs that are being actively secreted into circulation via exosomes, lipoproteins or protein complexes [50, 51]. There are several available methods for blood collection, storage, and RNA isolation, depending on the source of interest and the study design, for example: (1) PAXgene Blood RNA System, for collection of whole-blood (PreAnalytiX, Switzerland); (2) EDTA-Vacuette tubes, followed by centrifugation, to collect plasma or serum; (3) ExoQuick System for isolation of exosomes (System Biosciences, USA); or (4) LeukoLOCK Total RNA Isolation System, for isolation of RNA from WBCs (Life Technologies, USA). In this study, we used PAXgene tubes, which are intended for easy collection and transport, but more importantly, are optimized for the stabilization of RNA and long-term storage of blood samples. However, using PAXgene tubes makes it impossible to separate any of the blood fractions, thus allowing only the analysis of whole-blood. Although we did not test blood collection procedures or RNA extraction methods, the source of RNA and extraction method can have a significant impact on the measured levels of ncRNAs. Prichard *et al.* provides a comprehensive review on sample collection and processing for miRNA quantification [47].

The objective of this study is to provide researchers with general guidelines for quantification, data processing and analysis of miRNA, and other small non-coding RNAs (sncRNAs), from human clinical samples using NGS. Here, we test critical, alternative library preparation steps based on the ubiquitously used Illumina TruSeq small RNA sequencing methodology, as well as the effects of total RNA input and quality. Additionally, we describe methods for data processing, data analysis, and downstream validation techniques. Finally, we provide expression patterns and distribution of miRNAs and other sncRNAs from human whole-blood, brain, heart, and liver samples. This study provides tools and outcome metrics that will aid researchers and clinicians in choosing an appropriate quantification method, processing large amounts of data efficiently, and overall generating valuable information that can contribute to our understanding of small non-coding RNAs as potential biomarkers and mediators of biological functions and disease.

## Methods

### Human samples

A total of 45 biological samples were included in the present study, and include 1) peripheral blood samples (*N* = 32) obtained at a community outpatient clinic at the Douglas Mental Health University Institute from healthy anonymous volunteers; 2) postmortem, prefrontal cortex brain tissue (*N* = 4), which was obtained in collaboration with the Quebec Coroner’s Office and the Douglas-Bell Canada Brain Bank (Douglas Mental Health University Institute, Montreal, Canada); 3) commercially available, human brain (*N* = 1), human heart (*N* = 4), and human liver (*N* = 4) (Ambion). Ethics approval for this study was obtained from the Institutional Review Board of the Douglas Mental Health University Institute, and written informed consent was obtained from volunteers or family members, as appropriate.

### Sample processing and RNA extractions

Peripheral blood samples were collected in PAXgene blood RNA tubes (PreAnalytix, Switzerland). PAXgene tubes were frozen using a sequential freezing process. This involves storing tubes at room temperature for 3 h, transferring to 4 °C overnight, followed by 6–8 h at −20 °C and then final storage at −80 °C. Total RNA (including the miRNA fraction) was isolated from whole-blood using the PAXgene Blood miRNA Kit (Qiagen, Canada), according to manufacturer’s instructions. Furthermore, total RNA was isolated from frozen brain, heart and liver tissues using the miRNeasy Mini Kit protocol (Qiagen, Canada) with no modifications. RNA and miRNA yield and quality were determined using the Nanodrop 1000 (Thermo Scientific, USA) and Agilent 2100 Bioanalizer (Agilent Technologies, USA).

### Small RNA library preparation

All libraries were prepared using the Illumina TruSeq Small RNA protocol following the manufacturer’s instructions with 12 cycles of PCR amplification after ligation of the 3′ and 5′ adapters. This protocol is ideal for the investigation of small RNA species, as it takes advantage of the structure of most small RNA molecules by ligating specific adapters to the 5′-phosphate and 3′-hydroxyl group, which are molecular signatures of their biogenesis pathway. Individual libraries were prepared using a unique index primer in order to allow for pooling of multiple samples prior to sequencing. Experimental conditions were as follows:

1. *Comparison of small RNA library preparation methods* (Fig. 1a)

**Fig. 1:**
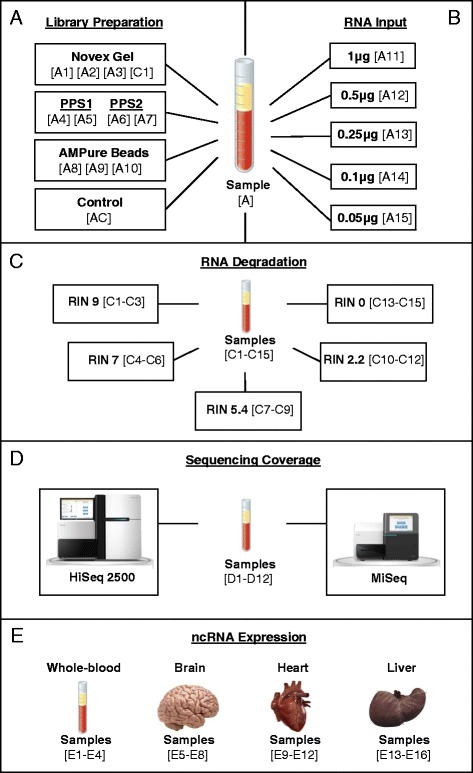
Illustration of study design and samples. Human biological samples (*N* = 45) were included in the present study. **a** Peripheral blood from a single individual was split into 11 aliquots (technical replicates) to test three different small RNA library purification methods: Novex TBE PAGE gel (*N* = 3), Pippin Prep automated gel system (PPS) (*N* = 4), and AMPure XP beads ((*N* = 3). Sample C1 (control-human brain) (*N* = 1), sample AC (control-no purification method) (*N* = 1). **b** Peripheral blood from a single individual was split into 5 aliquots (technical replicates) to test optimal amounts of RNA input: (1 μg), (0.5 μg), (0.25 μg), (0.1 μg), and (0.05 μg). All libraries were purified using the PPS system. **c** Peripheral blood samples from 15 healthy volunteers (biological replicates) to test the effects of RNA integrity. Samples were split into 5 groups (*N* = 3) with average RIN values of 9, 7, 5.4, 2.2 and 0. All libraries were purified using AMPure XP beads. **d** Peripheral blood samples from 12 healthy volunteers (biological replicates) to test effects of sequencing coverage. Samples sequenced on both a HiSeq2500 (*N* = 12) and MiSeq (*N* = 12) Illumina sequencers. All libraries were purified using AMPure XP beads. **e** Human whole-blood (*N* = 4), brain (*N* = 4), heart (*N* = 4) and liver (*N* = 4) tissues to test expression and tissue specificity of small ncRNAs. All libraries were purified using AMPure XP beads
2. *Testing RNA input amounts for small RNA library preparation* (Fig. 1b)
3. *Exploring the effects of RNA quality on small RNA library preparation* (Fig. 1c)
4. *Testing sequencing coverage for small RNA sequencing* (Fig. 1d)
5. *Characterization of ncRNA expression patterns in four human tissues* (Fig. 1e)

### Library preparation methods

In order to compare three different small RNA library purification methods, we prepared 11 libraries starting with 1 μg of good quality total RNA (RIN >8). All libraries were prepared using total RNA extracted from peripheral blood of a single individual (Fig. 1a). The RNA was split into 11 aliquots and each was used as a technical replicate. In addition, we used total RNA from commercially available human brain as a library preparation control. Libraries were purified as follows:

1. *Purification by Novex TBE PAGE gel:* 50 μl of amplified cDNA from samples A1-A3 and C1 were loaded into a 6 % Novex gel and run for 80 min at 130–135 V. After cleaning the gel with RNase free water, a band was manually cut to contain all fragments sized 145–160 nt, corresponding to mature miRNAs and other regulatory small RNA molecules (Additional file 1: Figure S1).
2. *Purification by Pippin Prep automated gel system (Sage 3 %):* The Pippin Prep system (PPS) allows automatic selection of specified cDNA products. 25 μl of amplified cDNA from samples A4-A7 were loaded into a Pippin Prep machine. Furthermore, in order to test variability between machines, samples A4 and A5 were loaded into PPS1, while samples A6 and A7 were loaded into PPS2. Size selection was automated for products between 125 and 180 nt (Additional file 1: Figure S2).
3. *Purification by AMPure XP beads:* Biotinylated magnetic AMPure beads allow for selection of specified cDNA products bound to streptavidin. 50 μl of amplified cDNA from samples A8-A10 were mixed and purified two times with AMPure XP beads at a 1.8:1 ratio (beads:sample). This ratio allows for optimal selection of all products higher than 100 nt.

Libraries were validated and quantified using an Agilent 2100 Bioanalyzer High Sensitivity DNA chip and qRT-PCR with the KAPA library quantification kit (Kapa Biosystems, USA). Sample C1 (control-human brain) was not sequenced. All additional samples (A1-A10), as well as sample AC (control-no purification method), were sequenced.

### Total RNA input amounts

Next, we tested the optimal amount of total RNA input required to prepare small RNA libraries from peripheral blood samples. As previously done, we split total RNA from the same individual into 5 aliquots and each was used as a technical replicate. We prepared 5 additional libraries, starting with different amounts of RNA: A11 (1 μg), A12 (0.5 μg), A13 (0.25 μg), A14 (0.1 μg), and A15 (0.05 μg) (Fig. 1b). All 5 libraries were purified using PPS and validated using an Agilent 2100 Bioanalyzer High Sensitivity DNA chip and qRT-PCR with the KAPA library quantification kit.

### Effects of RNA integrity

We also explored the effects of RNA integrity on library preparation for small RNA sequencing. To address this issue, we selected peripheral blood samples from 15 healthy volunteers. These samples were collected and processed following the same protocols as previously described, but were selected based on varying RNA integrity number (RIN) values. These values represent the level of RNA degradation in the sample, where 10 and 0 are the highest and lowest quality scores, respectively. The 15 samples were split into 5 groups with average RIN values of 9, 7, 5.4, 2.2 and 0 (Fig. 1c). Small RNA libraries were prepared as previously described, validated and quantified using an Agilent 2100 Bioanalyzer High Sensitivity DNA chip and qRT-PCR with the KAPA library quantification kit.

### Small RNA sequencing coverage

Next we tested how sequencing depth affects the amount of information obtained from whole-blood samples. We prepared small RNA libraries using total RNA from an additional 12 healthy volunteers, as previously described. All 12 libraries were pooled and sequenced on both a HiSeq2500 and MiSeq Illumina sequencers (Fig. 1d).

### Small ncRNA expression in human whole-blood and brain

To characterize the expression and explore tissue specificity of small ncRNAs in human biological samples, we prepared 16 additional libraries from human whole-blood, brain, heart and liver tissues (Fig. 1e). Brain, heart and liver libraries were prepared with 1 μg of total RNA, purified using AMPure beads, validated and quantified using an Agilent 2100 Bioanalyzer High Sensitivity DNA chip and qRT-PCR with the KAPA library quantification kit.

### Sequencing data processing and analysis-Small RNA-Seq Pipeline

Samples were sequenced at the McGill University and Genome Quebec Innovation Centre (Montreal, Canada) and the European Molecular Biology Laboratory (EMBL), Genomics Core Facility (Heidelberg, Germany), using the HiSeq2500 or MiSeq Illumina sequencers with 50 nt single-end reads. All sequencing data were processed using CASAVA 1.8+ [52] and extracted from FASTQ files. Fastx_toolkit [53] was used to trim the Illumina adapter sequences. Additional filtering based on defined cutoffs was applied in order to obtain high quality data. These filters included: 1) Phred quality (Q) mean scores higher than 30, 2) reads between 15–40 nt in length, 3) adapter detection based on perfect-10 nt match, and 4) removal of reads without detected adapter. Any specific cutoffs used in our small RNA sequencing pipeline can be adjusted according to any experimental design. For instance, one can choose to lower the Q score filtering criteria, loosen the adapter detection perfect-match, or decrease the size selection range. Nevertheless, there is a risk of introducing sequencing error probabilities or background noise to the data. Additionally, we used Bowtie [54] to align reads to the human genome (GRCh37) [55] and ncPRO-seq [56] in combination with miRBase (V20) to match them to known miRNA sequences [57, 58]. We used the Rfam [59] and NCBI’s piRNA [60] databases to map other small RNA sequences. Furthermore, all sequencing data was normalized with the Bioconductor–DESeq2 package [61], using a detection threshold of 1 count per miRNA (present at least once in each of the libraries tested). All RNA sequencing data used in this study is available on the NCBI-Gene Expression Omnibus database with accession code **GSE69825**.

### Quantitative Real-Time Polymerase Chain Reaction (qRT-PCR)

Small RNA sequencing data was validated using qRT-PCR. Total RNA samples were reverse transcribed using TaqMan RT-PCR microRNA assays (Applied Biosystems) according to the manufacturer’s instructions. Real-time PCR reactions were run in quadruplicate using the ABI 7900HT Fast Real-Time PCR System and data was collected using the Sequence Detection System 2.4 (SDS) software (Applied Biosystems). Expression of miRNAs was quantified using miRNA TaqMan probes (Applied Biosystems) and calculated using the Absolute Quantitation (AQ) standard curve method. RNU6B was used as an endogenous control as it showed expression levels that remained relatively constant with low variance and high abundance across the samples tested.

### Data analysis

All numerical data are expressed as the mean ± s.e.m. Statistical differences among groups were analyzed by Student’s t–test, One–Way ANOVA with post–hoc correction, and Pearson’s correlation coefficients. Statistical significance was calculated using GraphPad Prism5 and SPSS 20. *P* <0.05 was considered statistically significant.

## Results and Discussion

This study assessed several steps involved in the development of an ncRNA biomarker discovery pipeline, ranging from sample preparation to bioinformatic processing. At each stage, we evaluated the pros and cons of different techniques that may be suitable in some circumstances but not others, depending on experimental design. Evaluation methods included quality of data output in relation to hands-on laboratory time, cost, and efficiency of processing.

### Bioinformatic output measures for small RNA sequencing quality control

There are several important parameters to test in order to establish a high-throughput biomarker discovery pipeline including quality of the sample, library preparation methods, input quantity, and sequencing coverage. However, prior to testing these parameters, we established a set of output measures to allow us to compare across methodologies and experimental conditions. These quality control measures are described in detail on Table 1. In addition, we tested and compared our bioinformatics pipeline both internally (collaborators) and externally (online published available data) before analyzing any of the libraries in this study. Our findings were consistent with published results [51, 50, 62, 63].

**Table 1:** Bioinformatic output measures for small RNA sequencing quality control

QC metric	Description
Raw Reads	According to Illumina guidelines for small RNA sequencing, 1–2 M reads is an accepted range for expression profiling experiments, while 2–5 M reads is the accepted range for discovery applications.
Size	To avoid background noise due to small fragments of degraded RNA, we removed all reads <15 nt. Size filtering can be easily modified to target a specific small RNA species. For example, 15–28 nt (miRNAs), 24–31 nt (piRNAs), or 15–40 nt if interested in all small ncRNAs.
Quality	Quality (Q) is based on a Phred score, which estimates sequencing error probabilities per base. A Q = 10 means a 1/10 probability of incorrect base calling or 90 % accuracy; Q = 20 (1/100; 99 %); Q = 30 (1/1000; 99.9 %); and Q = 40 (1/10000; 99.99 %). We removed reads with a quality score <30.
Adapter-Adapter	Adapter detection can be adjusted to allow for one or more mismatches in the first 10 nt to identify and trim the adapters. In order to enhance high quality reads, we set our adapter detection threshold to a perfect-10 nt match. Ligation of the 3′ and 5′ adapters to each other happens by chance at a very low rate. However, this can become an important issue for libraries prepared from very small amounts of RNA. We removed all adapter-adapter reads.
RNAs > 40 nt	This feature refers to RNA reads larger than 40 nt in length. In most cases these reads map to midsize and larger non-coding RNA populations. The percentage of reads >40 nt can vary (1 %–50 %) depending on library preparation method used.
Surviving Reads	This metric shows the number of reads that pass all the quality and trimming filters previously described. A good quality library should have surviving rates between 50 % and 100 %, depending on method used.
Unmapped	Due to sequencing errors, stringent QC filters, or RNA from other species (usually added as control, i.e. PhiX), a very small percentage of reads do not map to any human genomic location.
Unique & Multi-Mapped	In contrast to other types of sequencing (DNA and larger RNA), the percentage of reads that map to multiple genomic locations in small RNA sequencing is expected to be high (>50 %). Several small RNAs are encoded at more than one genomic location. This is thought to be a compensatory mechanism or response to ncRNA knockouts by random mutations.
miRNA	We used miRBase to align our reads to known miRNA species. A high percentage of reads aligned to miRNAs is expected. However, this percentage can vary depending on the source and quality of RNA.
Other ncRNAs	Rfam and NCBI’s piRNA databases were used to map our reads to other small RNA species. The number of these reads is very small compared to miRNAs. However, just like with miRNAs, the number of reads mapping back to other sncRNAs is associated with the source and quality of RNA.
(Repeat, Coding gene, Unknown)	This refers to an additional portion of reads that map to repetitive sequences, coding genes, and unknown sequences in the human genome. The number of these reads is expected to be low.
miRNA Count	We set a detection threshold at one count per miRNA (present at least once in each of the libraries tested) in order to get a better picture of lowly expressed miRNAs. However, for quantification and discovery studies, we recommend higher detection thresholds, usually >10 or >20 counts per miRNA, to avoid background noise and false positives.

Important quality control (QC) measures for bioinformatic analysis of our high-throughput biomarker discovery pipeline

### Library purification methods of small RNA sequencing

First, we tested three commercially available library preparation methods for small RNA sequencing: (1) Novex TBE PAGE gel; (2) Pippin Prep automated gel system; and (3) AMPure XP beads (Fig. 1a). It is important to point out that the main goal of this experiment was not to single out the “best” purification method, but rather to test and highlight the strengths and limitations of the top available options and provide guidelines as to what would best fit a particular study design. We were able to obtain good quality sequencing libraries for all samples, but nonetheless, we found significant differences across purification methods.

Before purification, adapter-ligated libraries for all samples showed a peak corresponding to miRNAs around 147 nt in length (Additional file 1: Figure S3). After purification, all libraries showed a sharp, single peak, corresponding to miRNAs and other small non-coding RNA molecules (Additional file 1: Figure S4). Samples purified using a Novex TBE PAGE gel showed a sharp, single peak at 147 nt, corresponding to miRNAs and other small non-coding RNA molecules (Additional file 1: Figure S4a-c). The four libraries purified using PPS also showed single peaks corresponding to miRNAs, but these libraries contained more than 50 times more product after purification, as compared to the Novex gel method (Additional file 1: Figure S4E-H). Finally, samples purified with AMPure XP beads, showed similar results as PPS, but these libraries showed the additional presence of other small RNA molecules ranging from 160–225 nt in length (Additional file 1: Figure S4i-k). All libraries, plus a control sample (no purification), were pooled and sequenced in a single lane of the HiSeq2500.

**Fig. 2:**
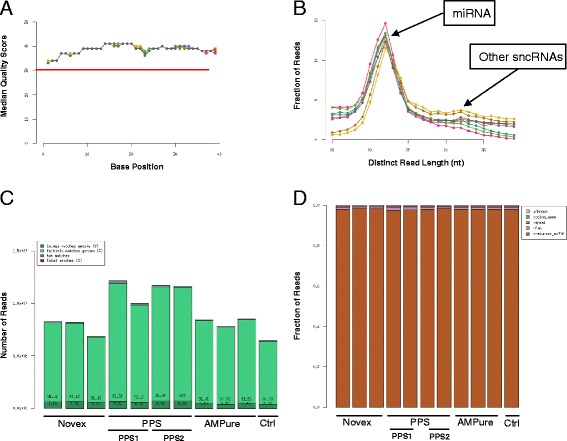
Quality control (QC) data (A1-A10). **a** Mean quality value scores over 40nts. **b** Distribution of reads based on length (19–25 nt, microRNAs) (30–35 nt, other sncRNAs). **c**-**d** Total number of reads, mapping percentages, and fraction of reads mapping RNA species

We obtained a total number of 109,956,847 raw reads, with a quality value of 37 in all libraries (Table 2 and Fig. 2a). The distribution of reads based on length showed a consistent pattern with a large peak between 19–25 nt and a small peak between 30–35 nt, corresponding to miRNAs and other sncRNAs (Fig. 2b). We found a significant difference in the total number of reads obtained depending on library purification method. Libraries prepared using PPS gave the highest number of total reads with an average of 11.8 M reads per sample, while with the others we obtained only an average of 8.8 M (Novex), 9.1 M (AMPure) and 8.5 M (no purification) (Table 2 and Fig. 2c). Purification method also had an effect on the total number of reads that survived our data processing and QC analyses. Only 3.13 % and 3.76 % of reads were removed using the Novex gel and PPS, respectively, while 25.22 % were removed when using AMPure XP beads. The no-purification control library showed similar results to those seen with AMPure beads. We then took a closer look at the reads that were removed and found small but significant differences across methods. For example, more reads were removed because they were smaller than 15 nt using AMPure beads (2.12 %). AMPure showed the highest quality of reads with an average of 1.2 % of reads removed as compared to Novex (1.6 %) and PPS (1.5 %). A very small number of reads were removed due to adapter-adapter ligation, but nonetheless, PPS showed the lowest percentage (0.03 %), while Novex, AMPure and control showed 0.05 %, 0.15 % and 0.53 %, respectively. The biggest difference across methods was explained by reads from RNAs >40 nt. Only a small number of reads were removed using Novex (1.1 %) and PPS (2.05 %), while an average of 21.71 % were removed when purifying libraries using AMPure XP beads (Table 2). Next, we looked at the portion of surviving reads. As expected, we found that more than 90 % of all reads mapped back to multiple locations in the genome, which is a defining characteristic of miRNAs [23, 45] (Fig. 2c). We did not find any significant differences in the number of reads that mapped back to known miRNA sequences across methods, with averages of 96.92 % (Novex), 96.45 % (PPS), 96.3 % (AMPure) and 96.24 % (control) (Table 2 and Fig. 2d). AMPure had the highest number of reads mapping back to additional small RNA molecules, while PPS contained the most reads mapping back to known repeat sequences in the genome. Finally, we found a significant difference in the average number of miRNAs identified: Novex (*N* = 415), PPS (*N* = 424), AMPure (*N* = 370) and control (*N* = 372).

**Table 2:** Purification method

Method	Novex	PPS	AMPure	Control
Sample	A1-A3	A4-A7	A8-A10	AC
Amount	1ug	1ug	1ug	1ug
RIN	8.2	8.2	8.2	8.2
Average Quality	37	37	37	37
Raw Reads	8.840869	11.871091	9.152952	8.491022
Size (<15 nt)	0.40 %	0.15 %	2.12 %	2.12 %
Low Quality (Q <30)	1.56 %	1.50 %	1.20 %	1.21 %
Adapter-Adapter	0.05 %	0.03 %	0.15 %	0.53 %
RNAs >40 nt	1.12 %	2.09 %	21.73 %	18.66 %
Surviving Reads	96.87 %	96.24 %	74.78 %	77.47 %
Unmapped	1.31 %	1.50 %	2.00 %	1.89 %
Unique-Mapped	7.21 %	6.44 %	6.52 %	6.42 %
Multi-Mapped	91.47 %	92.06 %	91.47 %	91.69 %
miRNA	96.92 %	96.45 %	96.03 %	96.24 %
Other ncRNAs	0.42 %	0.46 %	0.49 %	0.48 %
Repeat	0.77 %	1.04 %	0.88 %	0.82 %
Coding Gene	0.05 %	0.04 %	0.04 %	0.04 %
Unknown	0.52 %	0.48 %	0.41 %	0.42 %
miRNA Count (≥1)	415	425	370	372

Small RNA data analysis shows the percentage, composition and quality of reads from eleven libraries produced by our bioinformatics pipeline in order to test and compare three different small RNA library preparation methods

The Novex TBE PAGE gel proved to be the most specific for isolating the miRNA population in the samples. This is because we were able to manually and carefully cut the band between 145–160 nt corresponding to miRNAs from the gel and avoided any other smaller or larger RNA populations in the samples. However, we lost a significant amount of library product after purification from the gel, and in the end generated less reads after sequencing. In addition, this method requires a significant amount of hands-on time in the lab, which ultimately translates to very low throughput and significantly higher cost. We found purification by Novex gel to be a very good and specific method, particularly fit for small sample size projects where miRNAs are the main focus.

PPS generated the highest number both of total reads and distinct miRNAs identified, as well as very high specificity to miRNAs. This can be attributed to several factors, for example: (1) the libraries purified with PPS contained more than 50 times more product after purification, as compared to the Novex gel methods. This is due to the fact that the PPS is an automated system that does not require extraction of the library products directly from the gel, which can lead to less library product; (2) the range of the automatically isolated bands can be optimized to a desired product size (we used 125–180 nt), due to size selection and specificity, PPS contained the least number of reads removed due to a size either smaller than 15 nt or larger than 40 nt; (3) PPS showed the lowest number of adapter-adapter ligated reads. However, because each PPS instrument limits a run to only 4 samples, we tested variations across instruments. We found a significant difference in the final number of miRNAs identified per machine with 50 more miRNAs identified with PPS2. The PPS showed limitations in terms of consistency, and while the protocol requires less hands-on time in the laboratory, it does not increase throughput (only 4 samples per run) or cost significantly. We believe this is a very good method for medium size projects.

In contrast, purification by gel-free magnetic AMPure XP beads offers a high-throughput and consistent protocol. These libraries contained similar amounts of library product as compared to PPS (50X more than Novex). This method produced the highest quality of sequencing reads and the highest number of reads mapping back to other small RNA molecules. Nonetheless, because this method retained all products larger than 100 nt, AMPure beads produced the lowest specificity (to a single small RNA population), number of surviving reads and overall number of miRNAs (50 less than PPS). However, AMPure beads offer a very consistent, time efficient, high-throughput protocol with a significant reduction in labor time and cost. We found that purification by AMPure beads is a very good method, particularly fit for large projects where not only miRNAs but all small ncRNAs are the main focus. The results from this section are summarized in Table 3.

**Table 3:** Library preparation: purification methods

Method	Specificity	Throughput	Cost ($)	Study size
Novex TBE PAGE gel	High	Low	$$$$$	Small
	(manually cutting band; very specific)	(few libraries/day)		(2–10 samples)
Pippin Prep Automated gel system	Medium	Low	$$$	Medium
	(automated band; less specific)	(4 libraries/run [2 hrs])		(10–50 samples)
AMPure XP beads	Low	High	$	Large
	(all products >100 nt)	(24 libraries/2 hrs)		(50 and up)

Recommendations for small RNA sequencing library purification. Recommendations include: (1) Specificity: based on specificity to a particular small RNA population. (2) Throughput: based on the number of libraries that can be prepared per day and efficiency of processing. This number is relative to the number of people working and instruments available in the lab. (3) Cost: based on price of reagents, hands-on laboratory time, service fees by genome centers. (4) Study Size: based on number of biological or technical replicates

Finally, control sample AC was not purified or size selected before sequencing in order to compare the results to the three methods tested. However, all libraries in the study (including control sample AC) were prepared using the Illumina TruSeq Small RNA protocol. This protocol is ideal for the investigation of small RNA species, as it takes advantage of the structure of most small RNA molecules by ligating specific adapters to the 5′-phosphate and 3′-hydroxyl group, which are molecular signatures of their biogenesis pathway. This means that if the adapter ligation works well, in theory, the libraries don’t require any further purification. However, the success of purification methods also depends on suppression of adaptor dimer products in order to keep their representation at acceptable levels, ideally <2.5 %. The AC control results were similar to AMPure XP beads because, as previously explained, AMPure XP beads do not contain a very specific size selection (all products >100 nt) as opposed to Novex (145–160 nt) or PPS (125 and 180 nt).

### Total RNA input amounts for small RNA sequencing from whole-blood samples

Available amounts of starting RNA material are often a deciding factor when planning a study, as biological samples are often limited or hard to obtain. While planning an NGS project, there should be sufficient material set aside for an exploratory (profiling) experiment, as well as for technical validation and downstream experiments. We tested the feasibility of constructing good quality small RNA libraries from smaller amounts of RNA than the 1 μg suggested by Illumina (Fig. 1b). All libraries were processed as previously described and sequenced using the Illumina HiSeq2500. We obtained an average number of 11.6 M reads with an average quality score of 38 per library. Interestingly, we did not find any major differences or any significant correlation between amounts of starting RNA and any of the QC steps performed. Moreover, we found no significant differences in the number of surviving reads, reads mapping back to miRNAs, other RNA molecules, genomic repeats, unknown sequences or coding genes. In addition, the starting amount of total RNA had no significant correlation with the final number of miRNAs identified (Table 4). Our results suggest that good quality libraries for small RNA sequencing can be prepared with as little as 50 ng of total RNA from human whole-blood. This will reduce significantly the starting amounts of total RNA needed and will help preserve precious material for downstream experiments.

**Table 4:** Total RNA input

Sample	A11	A12	A13	A14	A15
Amount	1ug	0.5ug	0.25ug	0.1ug	0.05ug
RIN	8.2	8.2	8.2	8.2	8.2
Average Quality	38	38	38	38	38
Raw Reads	13.862726	7.995412	11.234898	11.921206	13.026487
Size (<15 nt)	0.12 %	0.11 %	0.54 %	0.18 %	0.29 %
Low Quality (Q <30)	0.99 %	1.02 %	1.11 %	1.04 %	1.22 %
Adapter-Adapter	0.02 %	0.03 %	0.13 %	0.13 %	0.17 %
RNAs >40 nt	0.75 %	3.40 %	1.05 %	1.33 %	0.91 %
Surviving Reads	98.12 %	95.44 %	97.17 %	97.32 %	97.41 %
Unmapped	1.64 %	2.17 %	1.93 %	1.99 %	2.11 %
Unique-Mapped	7.08 %	7.70 %	9.03 %	8.75 %	9.39 %
Multi-Mapped	91.27 %	90.14 %	89.03 %	89.27 %	88.51 %
miRNA	96.40 %	93.99 %	94.35 %	93.95 %	93.48 %
Other ncRNAs	0.47 %	0.78 %	0.84 %	0.88 %	0.95 %
Repeat	0.86 %	2.20 %	1.77 %	2.04 %	2.16 %
Coding Gene	0.05 %	0.07 %	0.09 %	0.09 %	0.11 %
Unknown	0.57 %	0.79 %	1.02 %	1.05 %	1.20 %
miRNA Count (≥1)	499	424	536	558	560

Small RNA data analysis shows the percentage, composition and quality of reads from five libraries produced by our bioinformatics pipeline to test RNA input amounts for small RNA library preparation

### Effects of RNA quality on small RNA sequencing

Clinical samples can be prone to RNA degradation due to methodological issues with sample collection and long periods of storage. In addition, RNA degradation has a significant impact on the profiling of messenger RNA (mRNA), which translates as background noise in the data. In most cases, these samples are not used or thrown away. However, several studies have shown that miRNAs display robust stability and are less susceptible to degradation [47, 64–69]. Here we designed an experiment to test the effects of RNA degradation on library construction and small RNA sequencing (Fig. 1c). From these libraries, we obtained an average of 12.9 M reads with an average quality score of 36. Surprisingly, we did not find any significant correlations between RIN and any of the QC metrics, nor with the number of miRNAs identified. Samples with the lowest RIN values had the highest percentage of reads removed due to size (<15 nt) and adapter-adapter ligation. However, the percentage of reads removed due to these effects was quite small and did not reach statistical significance (Table 5). Our results show that degradation (measured by RIN values) had negligible effects on our data. Moreover, these results confirm the robust stability of miRNAs in clinical samples, which makes accurate miRNA quantification with NGS feasible, even from clinical samples with low RIN values.

**Table 5:** RNA degradation: whole-blood

Sample	C1-C3	C4-C6	C7-C9	C10-C12	C13-C15
Tissue	Blood	Blood	Blood	Blood	Blood
RIN	9	7	6	2	0
Average Quality	36	36	36	36	35
Raw Reads	14.221591	15.528347	12.679709	14.225867	11.689436
Size (<15 nt)	3.78 %	4.92 %	3.99 %	3.54 %	6.23 %
Low Quality (Q <30)	2.82 %	2.96 %	3.00 %	2.63 %	3.42 %
Adapter-Adapter	1.11 %	0.47 %	0.38 %	0.85 %	3.35 %
RNAs >40 nt	25.56 %	21.41 %	28.98 %	23.04 %	15.47 %
Surviving Reads	66.73 %	70.24 %	63.67 %	69.95 %	71.53 %
Unmapped	3.26 %	4.30 %	3.53 %	2.81 %	3.40 %
Uniq-Mapped	7.78 %	8.83 %	6.82 %	7.98 %	7.74 %
Multi-Mapped	88.96 %	86.87 %	89.65 %	89.21 %	88.86 %
miRNA	91.57 %	87.64 %	92.01 %	93.66 %	89.99 %
Other ncRNAs	1.20 %	3.74 %	0.93 %	0.84 %	1.40 %
Repeat	2.52 %	2.57 %	2.29 %	1.64 %	3.25 %
Coding Gene	0.11 %	0.23 %	0.09 %	0.08 %	0.17 %
Unknown	1.35 %	1.53 %	1.15 %	0.97 %	1.79 %
miRNA Count (≥1)	469	463	399	476	488

Small RNA data analysis shows the percentage, composition and quality of reads from 15 libraries produced by our bioinformatics pipeline to test the effects of RNA quality on small RNA library preparation

### Sequencing coverage for small RNA sequencing

RNA sequencing coverage refers to the total number of reads to be sampled in a particular experiment and it is an important factor while planning NGS experiments. Coverage can have a significant effect on the quality of data, sensitivity of detection and overall cost of the project. Sequencing time and cost is considerably smaller when using a faster, lower-scale NGS platform. However, to our knowledge, there are no published reports that directly compare the levels and number of distinct miRNAs that can be measured from human blood using different scale sequencers, such as HiSeq and MiSeq. The purpose of this experiment was to determine thresholds for detectability of small ncRNAs and whether or not a fast-turnaround time sequencer like MiSeq can be used in biomarker discovery. Here, we sequenced 12 blood samples on both HiSeq2500 and MiSeq Illumina sequencers (Fig. 1d). We generated 138.7 M reads with an average of 11.6 M reads per library from the samples sequenced using the HiSeq2500 platform. As expected, using the same samples, we produced about 10 % the number of reads using the MiSeq platform, with a total of 10.7 M reads and average of 890 thousand reads per sample. Moreover, we found a very strong correlation in QC metrics and output across sequencing platforms, and did not find any significant differences. The average quality scores were 37 and 36 for HiSeq2500 and MiSeq, respectively. The main difference was found in the actual number of miRNAs identified. We found 563 distinct miRNAs using the HiSeq2500, while only 231 miRNAs with the MiSeq. This ratio was maintained when using different detection thresholds (i.e. >10 or >20 counts per miRNA in all libraries) (Table 6). We performed additional analyses to determine how many samples can optimally be pooled to help reduce the cost of sequencing, while still generating a decent amount of good quality data. We reduced each of the libraries, previously sequenced with HiSeq2500, by a factor of 2 in order to simulate doubling the number of samples pooled per lane. Based on our prior results, we predicted the number of distinct miRNAs that can be expected at different sequencing coverage (per million reads) in an average human blood sample. We found a 20 % decrease in the number of total miRNAs detected by doubling the number of samples per lane (from 12 to 24). That is, only under the assumption that increasing the number of samples by a factor of 2 decreases sequencing depth by an equal factor. However, we believe that the total number of reads is a better indicator of the total number of miRNAs that can be expected in a sample. Our results, summarized in Table 7, show the number of total miRNAs expected per million reads. These results provide important insight into sequencing strategies, time, and cost, and are particularly important when there is interest in lowly-expressed miRNAs.

**Table 6:** Sequencing coverage

Sample	D1-D12	D1-D12	Pearson (r)
RIN	7.4	7.4	-----
Sequencer	HiSeq2500	MiSeq	-----
Average Quality	37	36	-----
Raw Reads	11.556456	889645	-----
Size (<15 nt)	6.39 %	6.79 %	0.99039
Low Quality (Q <30)	1.68 %	1.33 %	0.95246
Adapter-Adapter	0.27 %	0.32 %	0.99639
RNAs >40 nt	37.63 %	33.13 %	0.98538
Surviving Reads	54.03 %	58.42 %	0.98834
Unmapped	6.01 %	5.61 %	0.99573
Uniq-Mapped	12.73 %	13.14 %	0.99653
Multi-Mapped	81.27 %	81.24 %	0.99672
miRNA	86.11 %	85.77 %	0.99374
Other ncRNAs	1.81 %	1.83 %	0.99512
Repeat	3.83 %	4.37 %	0.99679
Coding Gene	0.14 %	0.15 %	0.98360
Unknown	2.11 %	2.26 %	0.99109
miRNA Count (≥1)	563	231	0.99997
miRNA Count (≥10)	264	111	0.99997
miRNA Count (≥20)	217	92	0.99998

Small RNA data analysis shows the percentage, composition and quality of reads from 12 libraries produced by our bioinformatics pipeline in order to test sequencing coverage for small RNA sequencing. Libraries were sequenced on both on HiSeq2500 and MiSeq Illumina sequencers

**Table 7:** Number of total miRNAs expected per million reads in whole-blood

# of Reads (million)	12 M	6 M	3 M	1.5 M	1 M
miRNA Count (>1)	563	446	353	289	263
miRNA Count (>10)	264	216	177	138	124
miRNA Count (>20)	217	177	138	111	101

Number of total miRNAs expected per million reads at three different thresholds of detection

### Expression of miRNAs and other small ncRNAs in human biological samples

MicroRNA expression patterns can be tissue and cell specific. For example, miR-1 has been shown to be enriched in cardiomyocytes [70] while miR-122 is the highest expressed miRNA in the liver [71]. Others have shown that some miRNAs are uniquely present in specific body fluids, such plasma, tears, breast milk, and seminal fluid [72]. To explore this, here we sequenced 16 samples (E1-E16) using a MiSeq sequencer to compare the expression of small ncRNAs in four human tissues: whole-blood, brain, heart, and liver (Fig. 1e). We used miRBase, Rfam and NCBI’s piRNA databases to map miRNAs and other small RNAs.

**Fig. 3:**
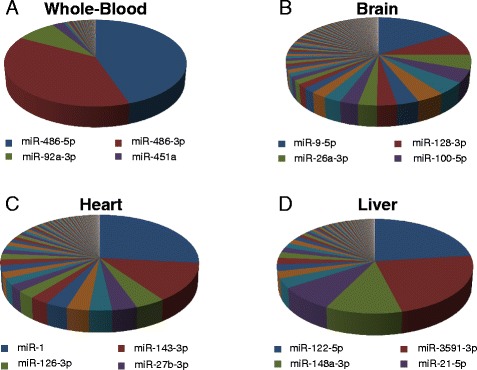
Expression of miRNAs in four human samples. Pie graph showing: **a** Whole-blood. **b** Brain. **c** Heart. **d** Liver

**Fig. 4:**
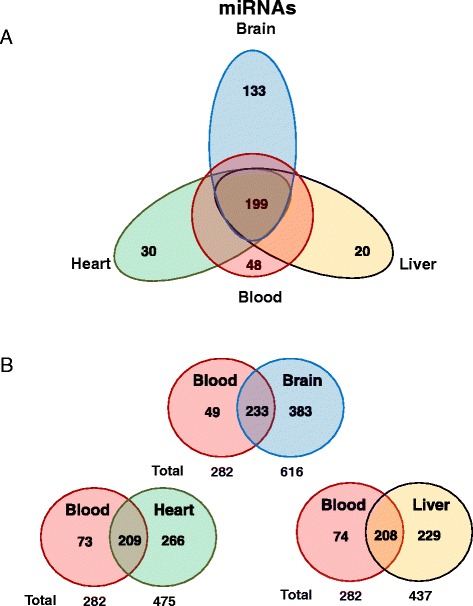
Tissue-specific patterns of expression of mi RNAs in human samples. Venn diagram showing: **a** Whole-blood vs. Brain vs. Heart vs. Liver. **b** Co-expression levels of miRNAs between whole-blood and other tissues

**Fig. 5:**
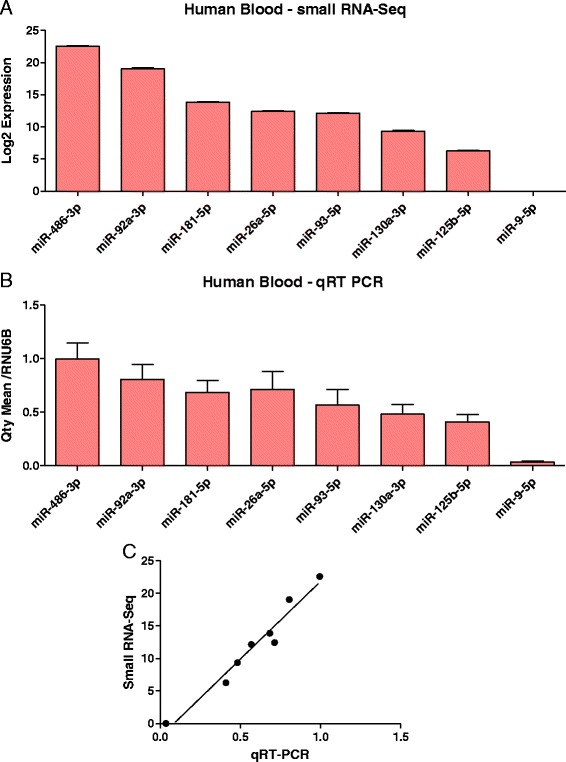
MicroRNA expression. **a** Bar graph showing small RNA sequencing Log2 expression of eight miRNAs in human whole-blood. **b** qRT-PCR validation. **c** Correlation of small RNA sequencing and qRT-PCR expression levels

We found tissue specific patterns of expression of miRNAs from these tissues. In whole-blood, the most abundant miRNAs were miR-486-5p, miR-486-3p which accounted for more than 90 % of all detectable miRNAs (Fig. 3a) (Additional file 2: Table S1). In human brain miR-9-5p, miR-128-3p, miR-26a-5p, miR-100-5p, and miR-99a-5p, made up 40 % of all detectable miRNAs (Fig. 3b and Additional file 2: Table S2). In human heart tissue, miR-1 accounted for more than 25 % of miRNAs, while miR-122 represented 23 % of all miRNAs expressed in human liver (Fig. 3c-d and Additional file 2: Tables S3–S4). Furthermore, human brain had the highest number of detected miRNAs, specifically 616, while heart, liver, and whole-blood had 475, 437, and 282, respectively. In addition, we found 48 miRNAs exclusively expressed in whole-blood, 133 only found in brain, 30 only in heart, and 20 only in liver tissue (Fig. 4a). We also explored the co-expression levels of miRNAs between whole-blood and the other tissues. Interestingly, we found 233 miRNAs co-expressed between whole-blood and brain (82.6 % of all miRNAs detected in whole-blood), 209 between whole-blood and heart (74.1 % of heart), and 208 between whole-blood and liver (73.8 % of liver) (Fig. 4b and Additional file 2: Table S5). To validate our miRNA sequencing results, we measured the expression of 8 miRNAs (miR-486-3p/92a-3p/181a-5p/26a-5p/93-5p/130a-3p/125b-5p/9-5p) with various levels of expression in peripheral blood using qRT-PCR. Our qRT-PCR results were consistent with our sequencing results (*P* <0.0001, Pearson’s r = 0.98, r^2^ = 0.95) (Fig. 5).

**Fig. 6:**
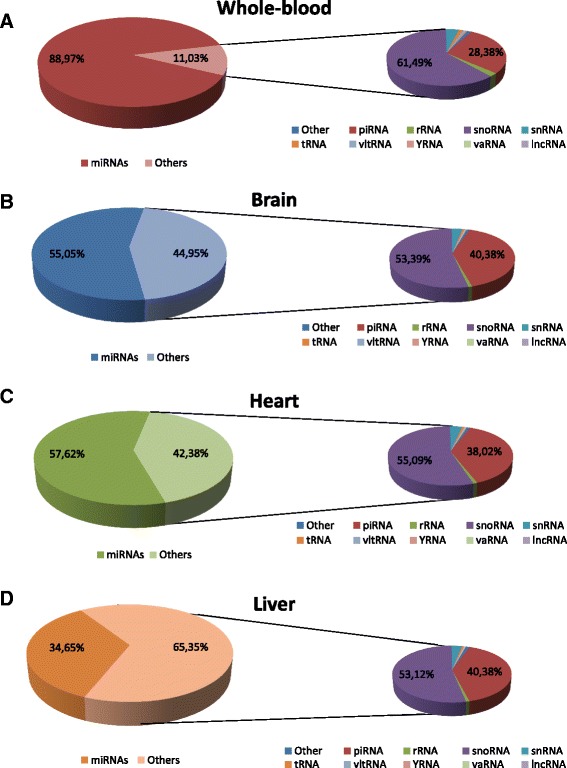
Expression and distribution of other small non-coding RNAs in four human samples. Pie graph showing: **a** Whole-blood. **b** Brain. **c** Heart. **d** Liver

**Fig. 7:**
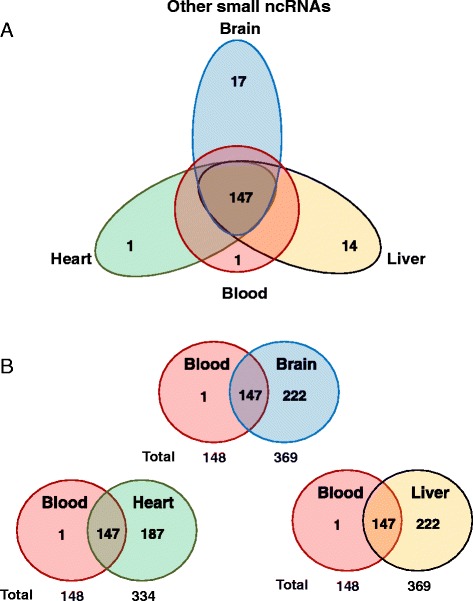
Tissue-specific patterns of expression of other small non-coding RNAs in human samples. Venn diagram showing: **a** Whole-blood vs. Brain vs. Heart vs. Liver. **b** Co-expression levels of miRNAs between whole-blood and other tissues

We also explored the expression of other small ncRNAs. In peripheral blood, we found an average of 11 % reads that mapped back to other species besides miRNAs. In other tissues we found more variation in the distribution of small RNAs. An average of 44.9 % of reads in brain tissue mapped back to other RNA species. Heart and liver showed 42.4 % and 65.4 % of reads mapping back to other small RNAs, respectively (Fig. 6). Brain and liver displayed the highest number of different small RNA molecules, both with 369, while blood and heart showed 148 and 334 respectively. In addition, 147 (out of 148) small RNAs expressed in whole-blood were co-expressed in all other tissues (Fig. 7a-b). The most abundant species of other small ncRNAs were snoRNAs and piRNAs, which made up more than 85 % across all tissues (Fig. 6). Among the other ncRNA molecules, we found small nuclear (snRNA), transfer (tRNA), ribosomal (rRNA), vault (vaRNA), viral (vRNA), Ro RNP associated Y RNA (Y-RNA), and short fractions of long non-coding RNAs (lncRNAs). The levels of expression and distribution of these molecules can be found in Additional file 2: Tables S6–S9. These results provide important insight into tissue specific expression and distribution of small ncRNAs, as well as co-expression levels between whole-blood and three other tissues (brain, heart, and liver).

## Conclusions

The goal of this study was to highlight some fundamental details of small ncRNA profiling, and provide the reader with general guidelines for quantification, data processing and analysis of sncRNAs from clinical samples using NGS. Our results show that good quality sequencing libraries can be prepared from small amounts of total RNA and that varying degradation levels in the samples do not have a significant effect on the overall quantification of sncRNAs via NGS. In addition, we discuss the strengths and limitations of three commercially available library preparation methods, describe our bioinformatics pipeline, provide recommendations for sequencing depth and coverage, and describe in detail the expression and distribution of all sncRNAs in four human tissues: whole-blood, brain, heart and liver. Ultimately, this study provides valuable information that will help researchers plan and execute future small RNA profiling studies that will contribute to the understanding of sncRNAs as potential biomarkers and mediators of biological functions and disease.

## Additional files

Additional file 1: Figure S1. Novex Gel analysis of small RNA libraries. Samples A1-A3 and C1. Illumina custom RNA ladder consists of three double stranded DNA fragments 145 bp, 160 bp, and 500 bp. The 147 nt band primarily contains mature microRNA generated from approximately 22 nt small RNA fragments. A second, 157 nt band containing piwi-interacting RNAs, as well as other regulatory small RNA molecules, is generated from approximately 30 nt RNA fragments. **Figure S2**. Purification by Pippin Prep automated gel system (Sage 3 %). The Pippin Prep system (PPS) allows automatic selection of specified cDNA products. 25 μl of amplified cDNA from samples A4-A7 were loaded into a Pippin Prep machine. In order to test variability between machines, samples A4 and A5 were loaded into PPS1, while samples A6 and A7 were loaded into PPS2. Size selection was automated for products between 125 and 180 nt. **Figure S3**. RNA sample trace of amplicons on High-Sensitivity DNA Chip. Before library purification, adapter-ligated libraries for all samples (A1-A10, C1 and AC) showed a peak corresponding to miRNAs around 147 nt in length. **Figure S4**. DNA 1000 Chip trace of the final libraries. After purification, all libraries (A1-A10, and C1) showed a sharp, single peak, corresponding to miRNAs and other small non-coding RNA molecules. Samples purified with AMPure XP beads (A8-A10), showed the additional presence of other small RNA molecules ranging from 160-225 nt in length. **Figure S5**. Heatmap plot of co-expressed miRNAs. MicroRNAs co-expressed between whole-blood and brain, heart, and liver. Additional file 2: Table S1. MicroRNA expression in human whole-blood. Small RNA sequencing raw reads showing expression, distribution and percentage of microRNAs identified in four human blood samples. **Table S2**. MicroRNA expression in human brain. Small RNA sequencing raw reads showing expression, distribution and percentage of microRNAs identified in four human brain samples. **Table S3**. MicroRNA expression in human heart. Small RNA sequencing raw reads showing expression, distribution and percentage of microRNAs identified in four human heart samples. **Table S4**. MicroRNA expression in human liver. Small RNA sequencing raw reads showing expression, distribution and percentage of microRNAs identified in four human liver samples. **Table S5**. Co-expressed miRNAs. MicroRNAs co-expressed between whole-blood and brain, heart, and liver. **Table S6**. Expression of other non-coding RNAs in human whole-blood. Small RNA sequencing raw reads showing expression, distribution and percentage of other small non-coding RNAs in four human blood samples. **Table S7**. Expression of other non-coding RNAs in human brain. Small RNA sequencing raw reads showing expression, distribution and percentage of other small non-coding RNAs in four human brain samples. **Table S8**. Expression of other non-coding RNAs in human heart. Small RNA sequencing raw reads showing expression, distribution and percentage of other small non-coding RNAs in four human heart samples. **Table S9**. Expression of other non-coding RNAs in human liver. Small RNA sequencing raw reads showing expression, distribution and percentage of other small non-coding RNAs in four human liver samples.
