# Supplementary material for: Biomarker discovery: quantification of microRNAs and other small non-coding RNAs using next generation sequencing
Source: BMC Med Genomics. 2015 Jul 1;8:35. doi: 10.1186/s12920-015-0109-x (PMC4487992; doi:10.1186/s12920-015-0109-x)

Supplementary Figure 1

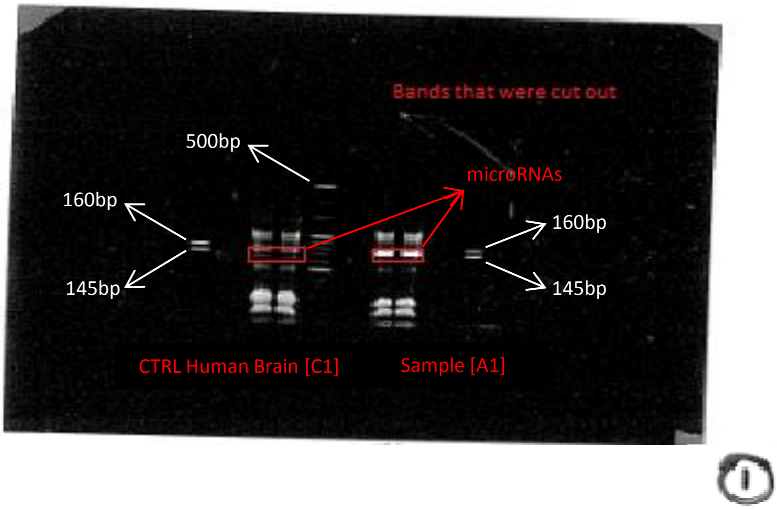

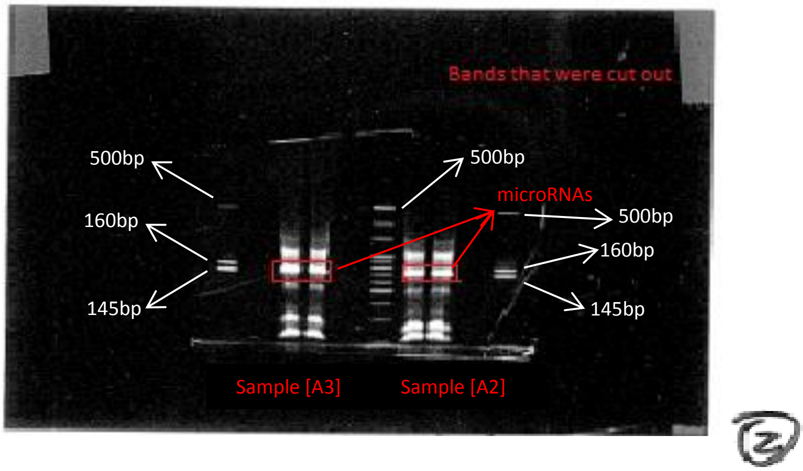

Supplementary Figure 2

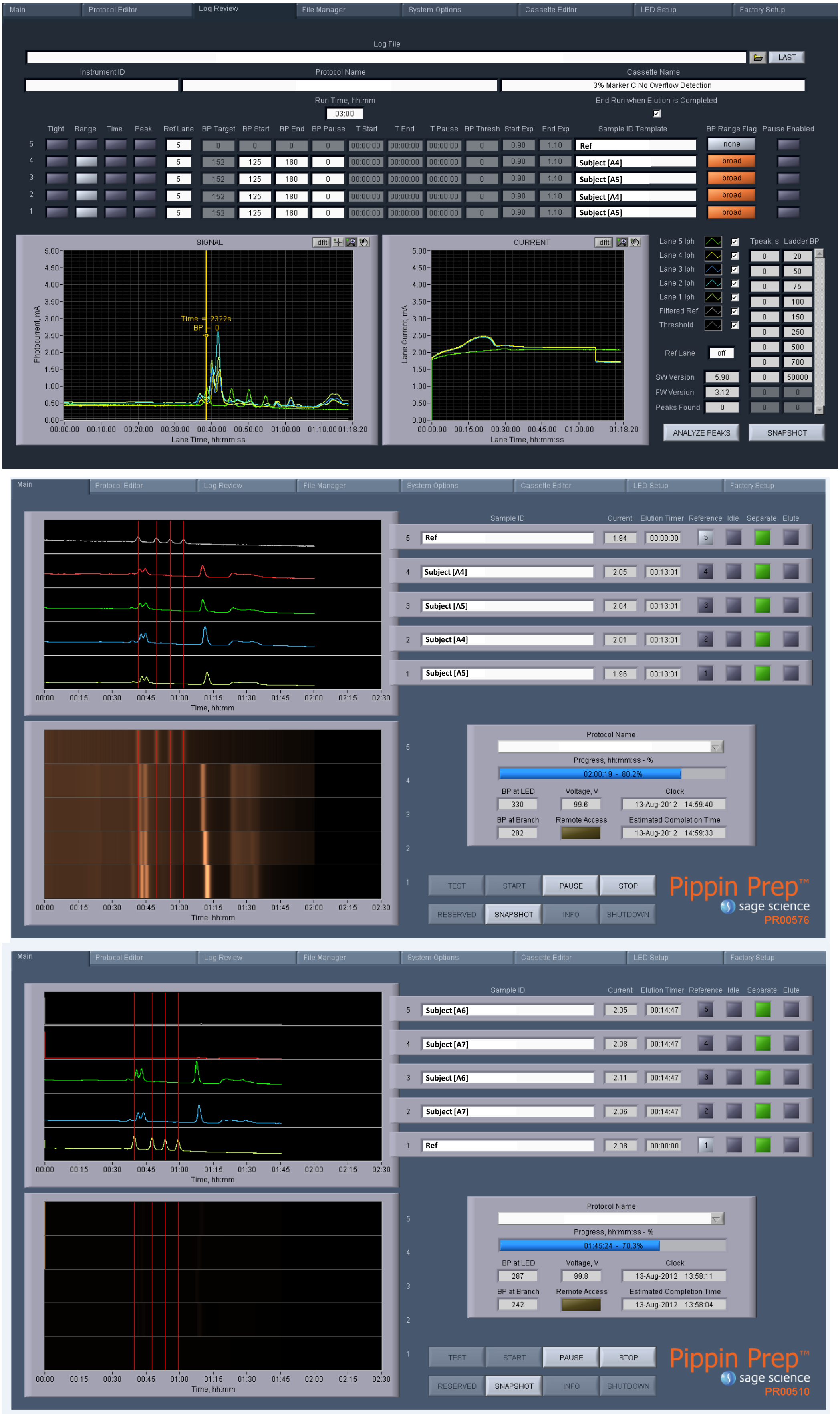

Supplementary Figure 3

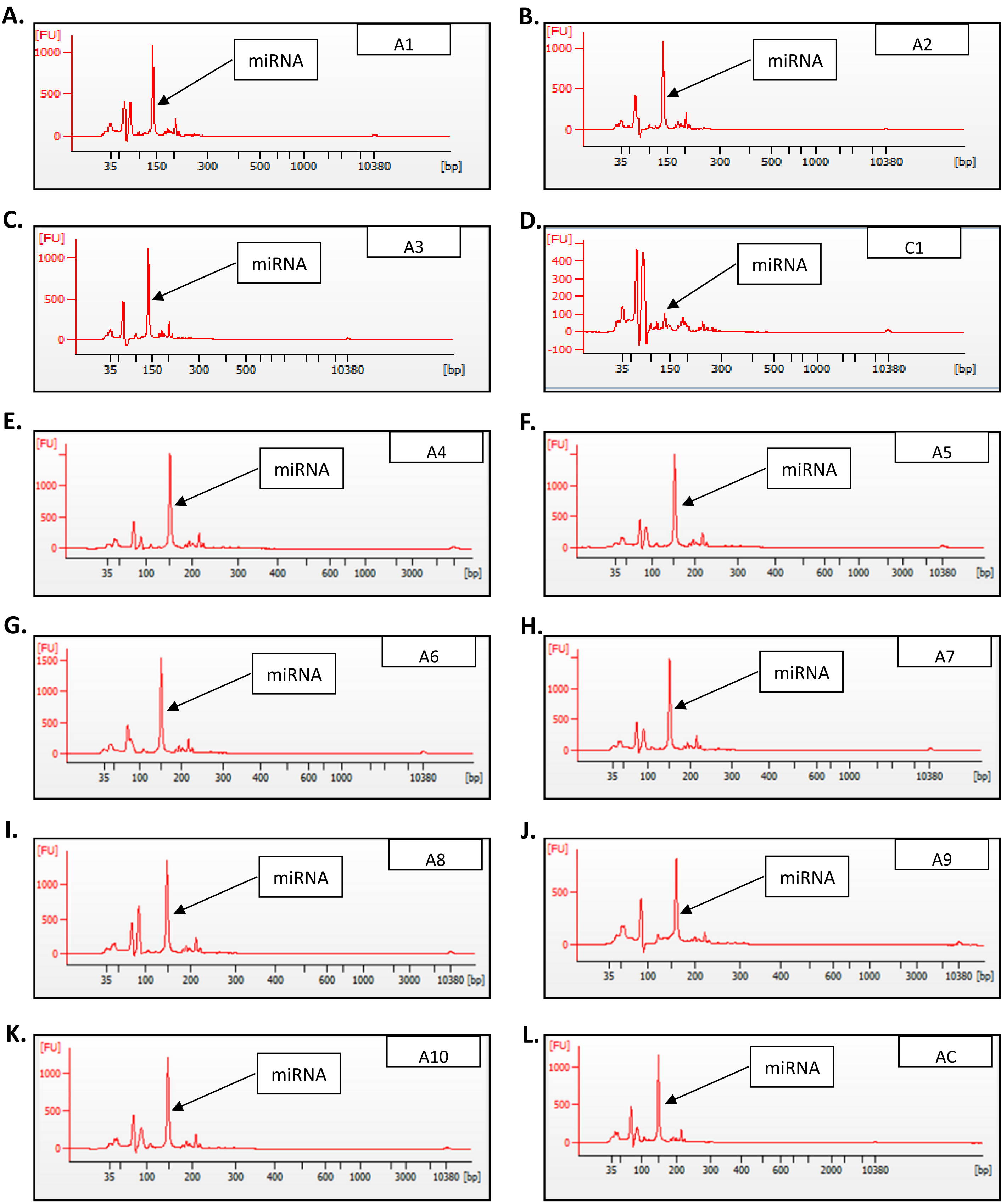

Supplementary Figure 4

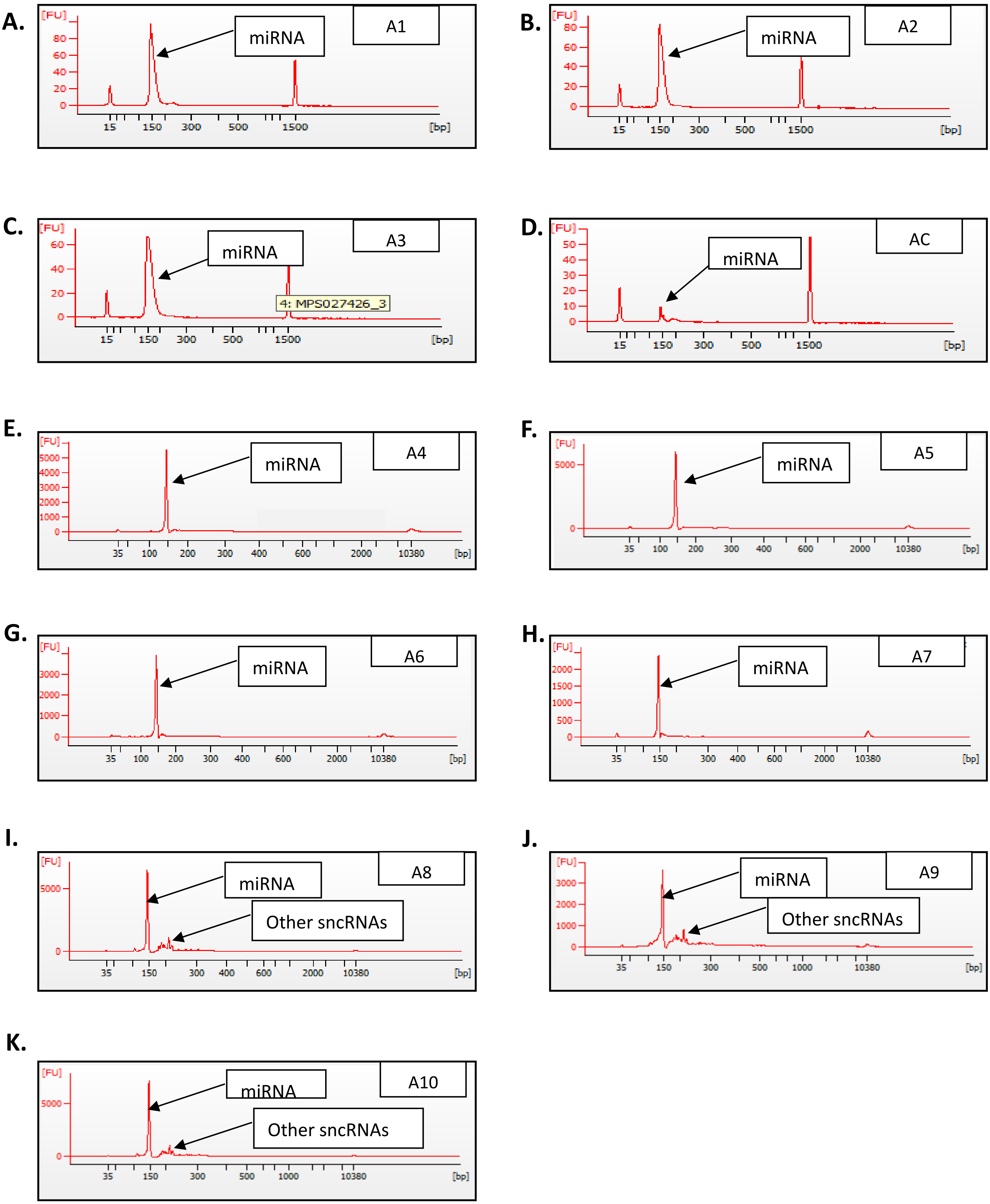

Supplementary Figure 5

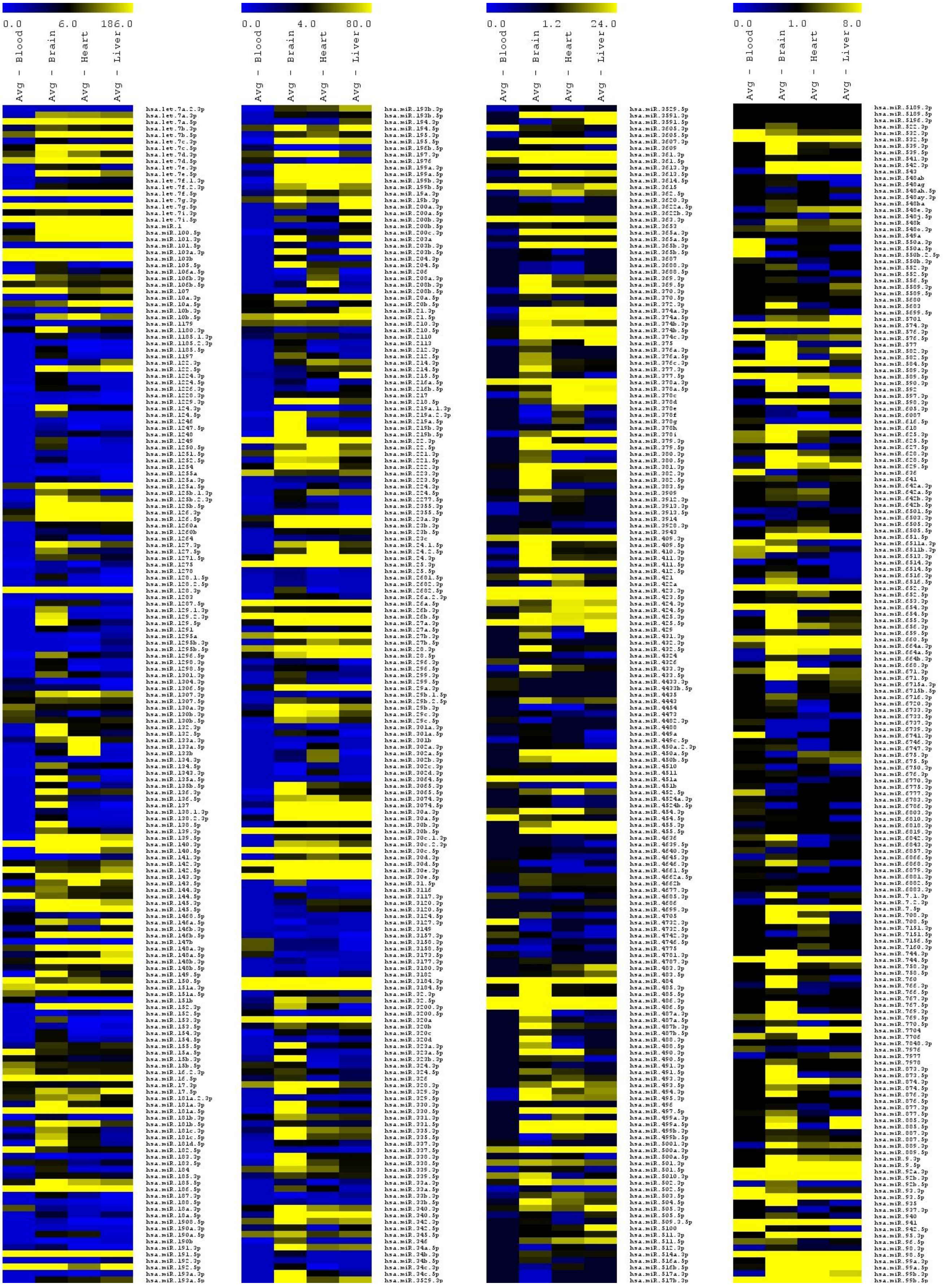

Supplement: Additional file 1: Figure S1. — Novex Gel analysis of small RNA libraries. Samples A1-A3 and C1. Illumina custom RNA ladder consists of three double stranded DNA fragments 145 bp, 160 bp, and 500 bp. The 147 nt band primarily contains mature microRNA generated from approximately 22 nt small RNA fragments. A second, 157 nt band containing piwi-interacting RNAs, as well as other regulatory small RNA molecules, is generated from approximately 30 nt RNA fragments. Figure S2. Purification by Pippin Prep automated gel system (Sage 3 %). The Pippin Prep system (PPS) allows automatic selection of specified cDNA products. 25 μl of amplified cDNA from samples A4-A7 were loaded into a Pippin Prep machine. In order to test variability between machines, samples A4 and A5 were loaded into PPS1, while samples A6 and A7 were loaded into PPS2. Size selection was automated for products between 125 and 180 nt. Figure S3. RNA sample trace of amplicons on High-Sensitivity DNA Chip. Before library purification, adapter-ligated libraries for all samples (A1-A10, C1 and AC) showed a peak corresponding to miRNAs around 147 nt in length. Figure S4. DNA 1000 Chip trace of the final libraries. After purification, all libraries (A1-A10, and C1) showed a sharp, single peak, corresponding to miRNAs and other small non-coding RNA molecules. Samples purified with AMPure XP beads (A8-A10), showed the additional presence of other small RNA molecules ranging from 160-225 nt in length. Figure S5. Heatmap plot of co-expressed miRNAs. MicroRNAs co-expressed between whole-blood and brain, heart, and liver. [file 12920_2015_109_MOESM1_ESM.pdf]
